# Search, reuse and sharing of research data in materials science and engineering—A qualitative interview study

**DOI:** 10.1371/journal.pone.0239216

**Published:** 2020-09-15

**Authors:** Bettina Suhr, Johanna Dungl, Alexander Stocker

**Affiliations:** Virtual Vehicle Research GmbH, Graz, Austria; University of Genova, ITALY

## Abstract

Open research data practices are a relatively new, thus still evolving part of scientific work, and their usage varies strongly within different scientific domains. In the literature, the investigation of open research data practices covers the whole range of big empirical studies covering multiple scientific domains to smaller, in depth studies analysing a single field of research. Despite the richness of literature on this topic, there is still a lack of knowledge on the (open) research data awareness and practices in materials science and engineering. While most current studies focus only on some aspects of open research data practices, we aim for a comprehensive understanding of all practices with respect to the considered scientific domain. Hence this study aims at 1) drawing the whole picture of search, reuse and sharing of research data 2) while focusing on materials science and engineering. The chosen approach allows to explore the connections between different aspects of open research data practices, e.g. between data sharing and data search. In depth interviews with 13 researchers in this field were conducted, transcribed verbatim, coded and analysed using content analysis. The main findings characterised research data in materials science and engineering as extremely diverse, often generated for a very specific research focus and needing a precise description of the data and the complete generation process for possible reuse. Results on research data search and reuse showed that the interviewees intended to reuse data but were mostly unfamiliar with (yet interested in) modern methods as dataset search engines, data journals or searching public repositories. Current research data sharing is not open, but bilaterally and usually encouraged by supervisors or employers. Project funding does affect data sharing in two ways: some researchers argue to share their data openly due to their funding agency’s policy, while others face legal restrictions for sharing as their projects are partly funded by industry. The time needed for a precise description of the data and their generation process is named as biggest obstacle for data sharing. From these findings, a precise set of actions is derived suitable to support Open Data, involving training for researchers and introducing rewards for data sharing on the level of universities and funding bodies.

## Introduction and motivation

The availability of research data affects the work of every researcher to a greater or lesser extent. In 2014, one of the authors of this paper, B. Suhr, with background in mathematics started working in a new field, granular materials, and faced the problems arising from the lack of openly available data. In the literature, both experimental and computational data was published in processed form, but it was the raw data, which would have facilitated the own scientific work. As a consequence of unavailable research data, experiments were panned, conducted and paid for. The idea of Open Data, to make data findable, available, integrable and reusable (known as the F.A.I.R. principle), is currently receiving much support from politics and funding agencies, e.g. the European Union. In 2016, the national funding agency Austrian Science Funds (FWF) initiated the pilot programme “Open Research Data (ORD)” in order to create role models for the openness of research data. From the author’s experiences, this funding scheme was very attractive, and a project was funded, which included to generate and share all research data needed for a specific purpose: the validation of Discrete Element Models for granular materials. The project team had to deal intensively with non-technical aspects, such as research data management and sharing, which is rather unusual in comparable research projects in this domain. In the granted project, it was decided to publish research (experimental) data accompanied with classical research articles to minimise the needed work for the description of the data (experiments). Up to now, three article/data set pairs are published [1–6], including 3D scan data or direct shear tests and uniaxial compression tests of two types of railway ballast.

For the publication of the datasets zenodo.org was chosen, as here the long-term availability is ensured and all datasets can be part of a so-called “Community Collection”, possibly increasing the findability of the datasets.

As part of the project, open research data practices of scientists in the authors’ scientific domain, materials science and engineering were studied. Open research data practices are understood to include the following aspects: generation of data, search for data, reuse of data and data sharing. The chosen approach allows to study connections between the single aspects of open research data practices and, as one author is member of the investigated domain, to gain deep insights into domain specific features. A qualitative interview study was preferred to a survey and was identified as the most sensible approach for this research, the exploration of (open) research data practices in the field of materials science and engineering. During interviews respondents can tell their story freely and naturally, which leads to more detailed insights and a more comprehensive picture on the subject of investigation. In the formulation of research questions, the first point was to carefully examine the characteristics of research data in the field of materials science and engineering, leading to the first research question:

**RQ1** What are the characteristics of research data needed in materials science and engineering?

The second research question was aimed at finding out which scientists generated their own data (of what type) and how this data was stored. Only those scientists, who generated their own research data, had the option to share this data.

**RQ2** What types of research data are generated by the researchers and how is this data stored?

Data reuse is essential for researchers without the possibility of own data generation, but may also be interesting for those who generate their own data. Therefore, the next research question dealt with the topic of data reuse, including the researchers general attitude towards data reuse, their data search strategies and actual reuse behaviour.

**RQ3** What is the current state of data reuse in materials science and engineering?

Those researchers, who generate their own research data, do principally have the option to share this data. The fourth research question was formulated to find out if data is shared and the reasons why it is done or not.

**RQ4** How do researchers in materials science and engineering share their research data? Why/why not?

The next research question involved all researchers and their views on incentives and obstacles for data sharing.

**RQ5** What are incentives and obstacles for data sharing perceived by researchers within the materials science and engineering domain?

Research data practices are the subject of intensive research, which is reflected by a rich body of literature dealing with these different aspects. A brief literature review will be given in the following section. However, the materials science and engineering domain was not in focus in published research on research data practices. Obviously, research data practices will vary strongly among scientific disciplines, as e.g. climate research or genetics will be very limited without data sharing while e.g. health related research faces legal restrictions protecting the privacy of participants/patients. Hence, it is to be examined, how the materials science and engineering domain perceives the concept of open (research) data in general, which is in the focus of the sixth research question.

**RQ6** How established is the concept of Open Data in the field of materials science and engineering?

The investigation of these six research questions will provide a more complete picture of the current state of open research data practices in the field of materials science and engineering. The chosen approach allows to study the connections between the different aspects of open research data practices, e.g. how data characteristics complicate reuse and sharing. As one of authors is a member of the investigated domain, this insight will be furthermore used to gain a deeper understanding of obstacles and incentives for data sharing or reuse. The obtained results are the basis for a derivation of precise actions, which could support Open Data, and to formulate obstacles which will remain in the view of the authors.

This remaining paper is organised as follows: The next section gives a brief literature review on the different aspects of research data sharing. The methods used in this qualitative interview study are outlined in the following section. The next two sections contain the obtained results and their discussion. In the last section, conclusions are drawn, including potentials and obstacles for Open Data in the investigated field.

## Literature review

The possibility to share, search and reuse research data is a rather new concept, [7]. Researchers might benefit if they can reuse data generated by other researchers, but they are also affected by additional work resulting from data sharing, which can be required by funding agencies or publishers of certain journals. Moreover, it might be difficult for researchers to keep up with new developments both on technological side, e.g. dataset search engines, or on scientific publisher’s sides, e.g. data journals. Here, some key findings of studies dealing with the different aspects of research data practices will be summarised. Purely domain specific studies, e.g. [8] investigated data sharing of geophysicists or [9] investigated data sharing among environmental scientists, will not be discussed.

The mandatory prerequisite of data reuse is the search for research data. In [10], literature on data retrieval practices is presented. For selected disciplines, similarities in how users search for research data are identified. In [11], the same group of authors investigate the search for research data from a socio-technical perspective by combining results from the literature with conducted interviews.

A very recent phenomenon is the development of dataset search engines. Google started the beta version of “Google Dataset Search” in 2018. Considering the importance of “Google Scholar” for the search of classical research papers, “Google Dataset Search” could have a high potential to make finding datasets easier. More details as well as a discussion of pros and cons can be found in [12]. As dataset search engines are a very recent phenomenon, little research was published yet on researcher’s usage of such dataset search engines, e.g. [10] did not address the topic.

Another modern method to both search for/access data and share is the use of data journals. There exist pure data journals, which publish exclusively data papers, but also mixed ones, where also classic research articles are published. While many data journals are specific to a research domain/topic, among the three biggest (w.r.t. number of articles) are two data journals, which are open to all fields: Elsevier’s “Data in Brief” and Springer’s “Scientific Data”. The change in this area is addressed in [13], where the current state is compared to the one described in [14] from 2015. The number of data journals grows slower today, while the number of published data papers increases fast. However, the number of data papers in 2019 (11500) represents roughly 0.4% of all research publications in 2017, [13]. Although data journals introduce a peer-review process to the publication of data, this process is not as mature as it is for classical research articles, [15].

In 2013, [16] investigated the data sharing and reuse in the “long tail of science”. While the reported sharing and reuse practices can be expected to have changed over time, the used definitions big/small science will be adopted in the current work. [16] describe that “Data from big science (large teams, long-term projects, extensive instrumentation) may be great in volume but usually are consistent in structure.” In contrast, in “the long tail of science, individuals and small teams collect data for specific projects. These data tend to be small in volume, local in character, intended for use only by these teams, and are less likely to be structured in ways that allow data to be transferred easily between teams or individuals.” [16] provide also references that small science “constitute the major portion of scientific funding”.

In 2015, Tenopir et al. [17] used big surveys to compare the state of data sharing and reuse perceptions with the results they obtained in 2011, [18]. They found that researchers’ data sharing behaviour is increasing but there are also perceived risks and barriers that might slow down this process. Investigating differences across age, geographic, and discipline-based groups they found that relevant issues were based more on cultural and discipline-based differences than on age. So, researchers “who work with human subjects were significantly less willing to share their data than respondents other disciplines. This may be attributable to the sensitive nature of protected health information with which they work”, [17]. In [19] data reuse is considered exclusively. The authors investigated the relation of researchers’ attitudes towards data reuse and their actual reuse behaviour. A greater reuse was found to correspond to the perceived importance of data reuse as well as its perceived efficacy and efficiency. “Expressed lack of trust in existing data and perceived norms against data reuse were not found to be major impediments for reuse contrary to our expectations”, [19]. In [20], a multilevel analysis was combined with an integrated theoretical framework to investigate discipline-based differences in researchers’ data reuse behaviour by “considering their disciplinary environments and individual motivations together.” It was stated that researchers’ intended reuse behaviour was influenced through their disciplinary environments, e.g. the availability of data repositories, as well as individual motivation: perceived usefulness, perceived concern and the availability of internal resources. For a further facilitation of data reuse three steps are suggested: “Educating scientists, providing internal supports, and providing external resources and supports such as data repositories”, [20].

In 2019, Chawinga and Zinn, [7], published an extensive literature review on data sharing behaviour, including more than 100 papers. They investigated which factors either supported or hinder data sharing on an individual, institutional or international level. At the individual level three main factors were reported to restrict data sharing: lack of time (for data preparation, description and actual sharing), researchers’ interest to remain control over “their” data and researcher’s fear of data misuse. At institutional level, three factors for supporting data sharing were identified: training of researchers in data sharing, compensation for data sharing (e.g. similar to compensations for classical research papers) and organisational policies encouraging data sharing. At international level data sharing policies of funding agencies and journal publishers can positively influence researchers’ data sharing behaviour. As an example, [21], Federer et al. investigated the PLOS ONE journal and the effect of its policy requiring researchers to share the data used in their publication. In the considered time period (between 2014 and 2016) the number of papers including a data availability statement increased but only 20% of all paper did share their data in a repository, which is the preferred method. Federer et al. suggest more stringent policies to further increase data sharing.

The citation of datasets remains an evolving issue with some open points. For example, in [22], Silvello addresses three problems. First, identification, e.g. of single, subsets or aggregated resources. Second, completeness, e.g. citing extracted data of large, evolving databases. Third, fixity, i.e. to guarantee access to the cited data. Apart from such problems, the questions is if researchers do formally cite the datasets, which they use. In an empirical investigation, Zhao et al. [23], analysed dataset mentions and citations in 600 publications in PLOS ONE. Unsurprisingly, big variations between different scientific fields were found, regarding dataset generation, reference and curation. It was stated that for most papers, there was a free access to the data, but “formal ways of data attribution such as DOIs and data citations were used in a limited number of articles”, [23]. From the results presented in [23], it seems that some researchers might miss to correctly cite the datasets they reused (although this can hardly be quantified in such an analysis). One possibility to solve this problem is presented in [24], where scientists develop a framework, which allows to find links between papers and datasets, identifying cases where a data citations might be missing. Moreover, a standard for measuring and displaying data user metrics is worked on. In [25], Parsons et al. review the history and future of data citations. “We know how to cite most data in research publications. We must only accelerate the implementation, and there does appear to be movement in that direction”, [25].

The literature review has revealed that there is already broad scientific knowledge on various aspects of open research data practices, also related to search, reuse and sharing of open data. There are many scientific publications that deal in depth with examining one or more of these practices applying different research methods. However, there is a lack of knowledge when it comes to exploring the awareness of open research data and (open) research data practices within specific domains on a more comprehensive level, which especially is true for the materials science and engineering domain.

## Methods

### Study design

To investigate open research data practices in the field of materials science and engineering, a qualitative research approach was chosen including semi-structured interviews. The study design was developed according to the “Consolidated criteria for reporting qualitative research (COREQ): a 32-item checklist for interviews and focus groups”, [26]. For conduction of the interviews, the authors developed an interview guideline, see the supplemental material provided with this work. A. Stocker, who has a PhD in Information Science, reviewed the literature on open research data practices. B. Suhr has a PhD in Mathematics, works in materials science and engineering and contributed practical experience in research data search and reuse. This knowledge was synthesised into the interview guideline. The guideline started with questions regarding the researcher’s scientific background, career stage etc. and then explored research data needs, search for research data and research data usage. The next important points were data generation, collection and sharing practices of the researchers. The interview guideline concluded with questions regarding research data management and knowledge/attitudes towards Open Data and Open Science in general. The interview guideline was formulated using several open-ended questions, to encourage a detailed discussion on the topic. Also, the researcher’s understanding of the term “research data” was subject of the interview. A pilot interview was conducted to test the interview guide and small adaptations were made. The pilot interview was not included in the analysis.

### Study participants and recruitment

The recruitment of researchers for the interviews was not easy. In the field of materials science and engineering, scientists rarely come into contact with qualitative interview studies. The authors of this study considered it unlikely to be able to convince strangers to take part in an one hour interview. Therefore, it was decided to contact researchers from the professional network of B. Suhr and ask for participation in this study. Out of 20 contacted researchers, 13 agreed to take part in the interviews. The choice of the participants was independent from their opinion towards data sharing. Researchers with negative views/no experience on the topic were particularly encouraged by the authors to take part in the interview, which was not successful in one case. In this way, the authors aimed at getting a more complete view on data sharing practices and attitudes within the domain, despite the small number of conducted interviews. The Open Data topic is often investigated using surveys of thousands of researchers, e.g. [17, 27]. As it is criticised in [28], such studies could possibly suffer from selection bias. [28] states “Researchers who are not concerned with the promotion of data access would logically be more likely to skip this survey, thus skewing the results in the direction of increased favourability towards sharing.” This problem is not easily addressed, no matter whether big surveys are conducted or small-scale interviews with detailed discussions. In both cases, the knowledge on structural problems should be integrated in the interpretation of obtained results. Other researchers, who chose not to participate in the interview, either named a lack of time as a reason or did not answer to two mails with the interview invitation.

Details on the interviewed researchers are summarised in Table 1. From the 13 interviews conducted, 12 participants were male and one female. The interviewed researchers had between 4 and 30 years of experience and their scientific career stage varied from PhD-student to university professor. Four researchers were employed at research centres and nine at universities. They were located in five different European counties (Austria, Italy, Netherlands, Spain and United Kingdom) and one in China. The educational background from the interviewees showed quite a big range, with the majority of participants having a degree in Engineering (including Civil, Geophysical, Industrial, Chemical and Mechanical Engineering). Two researchers had a degree in Materials Science and one in Mathematics. As the interviewees were chosen from the authors’ professional network, the educational background is not included in Table 1 to avoid the identifiability of single persons. The spectrum of educational background shows that the characterisation of the scientific domain is not easy. Most of the researchers work with granular materials, which is part of materials science and engineering.

**Table 1:** Participant description.

no.	gender	career stage	year of experience	employer	country of employment
1	M	postdoc	10	research center	Austria
2	M	postdoc	7	research center	Austria
3	M	postdoc	23	research center	Austria
4	M	lecturer	5	university	UK
5	F	postdoc	8	university	Italy
6	M	PhD student	4	university	UK
7	M	professor	10	university	Netherlands
8	M	postdoc	5	university	Netherlands
9	M	postdoc	7	research center	Spain
10	M	PhD student	5	university	China
11	M	professor	30	university	Austria
12	M	professor	12	university	Austria
13	M	postdoc	7	university	Austria

In qualitative interview studies, the purposeful sampling technique, as described in [29], is frequently applied. This technique is a non-probabilistic sampling method, which ensures that different characteristics are covered by the interview participants. Due to the described problems with recruitment, this method could not be applied. However, the participants’ characteristics cover the complete range of scientific career stages and years of experience. Also, both universities and research centers are present as employer. It was clearly not possible to cover both genders, due to the low number of women being part of the professional network and working in the field of materials science and engineering. The authors would have preferred to cover a wider range in the country of employment, but at least five European countries are present and also China. Thus, although no purposeful sampling could be applied, most characteristics are well covered by the participants. The choice of the sample size is always difficult for qualitative interview studies, as no standard exists. The question of sample size is frequently connected with the term of saturation. Initially, [29] introduced theoretical saturation in the approach of grounded theory with a precise meaning. As mentioned in [30], this concept was later termed data/thematic saturation in other qualitative methods and here its meaning in less developed. According to [30], saturation is sometimes understood that data should be continuously collected until nothing new is generated. After a discussion of related problems, [30] concluded that “adopting saturation as a generic quality marker is inappropriate”. In [31], the concept of information power is introduced for choosing sample size in qualitative interview studies. When the sample holds more information, which are relevant for the current study, than less participants are needed, according to this concept. A model including five influential factors was developed, which indicate if a sample size should be rather large or rather small (not indicating absolute sample sizes but as recommendation for systematic recruitment). In our study, 13 interviews were conducted. Applying the information power influence factor model of [31], three factors reduce the needed sample size: the study’s aim was narrow (dealing with a special scientific discipline only), the sample specificity was dense (“participants who belong to the specified target group while also exhibiting some variation within the experiences to be explored”, [31]) and the quality of dialogue was strong (all interviews were conducted by J. Dungl, whose scientific background is communication studies). One factor of the information power model indicated that a rather large sample size is needed: the cross-case analysis strategy. The last factor is the application of established theory, which is ambiguous for this study: for the sampling no established theory could be applied (due to the problems described above), while this was the case for the analysis. With three factors indicating a smaller sample size, one factor indicating a larger sample size and one ambiguous factor, the literature was searched to check the sample sizes of comparable qualitative interview studies on (aspects of) open data practices of researchers of a specific domain. Seven works were found in the literature: data sharing of natural resources and environmental scientists was investigated in [9] with six interviews, data sharing of crop scientists was studied in [32] with seven interviews, 13 interviews were conducted with researchers of a special funding scheme in [33], also 13 interviews were conducted in [34] to study data reuse of social scientists, data practices of agricultural scientists were investigated with 14 interviews in [35], 20 interviews were conducted with researchers working in “small science” interested in data management or sharing in [36], and data reuse in archaeology was studied with 22 interviews in [37]. With the presented theoretical basis for choosing the sample size and the sample sizes of comparable studies ranging from six to 22, the sample sizes of 13 of the current study is considered as justified.

### Data collection and analysis

All interviews were conducted by J. Dungl, whose scientific background is communication studies. She had no former established contact with the participants apart from scheduling the interview. All interviews took place in June and July 2019. The interviews were conducted either in person or via telephone and the typical interview time was about an hour. All participants received oral and written information from the interviewer about the research aim and procedures and provided written informed consent. As already mentioned, the interview guideline contained several open-ended questions to encourage detailed discussions, e.g. “How do you identify organisations or people that may have data that could be useful to you?”. Moreover, the interviews were semi-structured, which allowed the participants to bring up own points in the interviews. In addition to this, also several closed questions were part of the interview, to investigate if researchers were familiar with certain aspects, e.g. “Do you use dataset search engines (e.g. Google Dataset Search)?”. All interviews were audio recorded and from this a transcript was written using the software “Transcriber”. The obtained transcripts were loaded in the qualitative data analysis software “QDA Miner Lite” for coding and analysis. Following qualitative analysis methods, see e.g [38], data was reduced and displayed before conclusions could be drawn. After all interviews were conducted, the interviewer developed a first coding scheme. This coding scheme was thoroughly discussed with all authors. In an iterative approach, the coding was adapted until a consensus on the used coding was reached. The results section contains also four figures, where used codes and categories can be seen. All quotes presented in this work were send to the interviewees for authentication.

## Results

### Results RQ1: Domain specific needs for research data

The interviewees were asked, which data they need to conduct their research. The answers could be classified as *experimental data* and/or *computational data*, which is in agreement with classifications used in [10].

Only a few of the interviewed researchers stated that they need *computational data* to conduct their research. This notion was not always explained in more detail, one researcher stated to need

“*these data in order to validate or to calibrate the theoretical model*”

P5

while another one referred to material parameters in general and knowledge on algorithms used in computations. Thus, it seems for the notion of computational data some standardisation of the word itself might be needed.

All of the interviewed researchers stated to need experimental data, but the data they named was very diverse, including recorded forces, paths, velocities as well as images, and depended strongly on the research focus of the interviewees. For demonstration, two example types of data will be named, which were mentioned by four researchers each, all of them working on granular materials.

1. geotechnical tests for the characterisation of the mechanical bulk behaviour of the granular material, i.e. triaxial tests, direct shear tests, oedometric tests
2. data on the shape of single grains, e.g. 3D meshes derived through 3D laser scanning, computer tomography or X-ray

The geotechnical tests are traditional measurements used for decades for this purpose. Although for some of them technical norms exist, describing the experimental conduction, e.g. [39], there exist no standardised way for measurement data description, i.e. no metadata standard. Compared to the geotechnical tests, research on grain shape is a more recent phenomenon, which is due to availability/development of measurement devices and computational resources. Most likely new research trends will generate/need different types of research data, possibly measured using newly developed devices. It can be expected that this will provide a big challenge for a research domain, which seems to be slow in developing standardised data description methods. Although these data description standards do not exist, researchers state the importance of a detailed description of the measurement situation. One big problem is missing information, which is addressed by two researchers:

“*I would really need the raw data, actually, to really compare it (to my research), but many a times it is not only the data that is missing, but also the how the experiments were performed*.”

P2

“*In order for such a thing to be really useful for future research, one would have to describe the origin of this data much more precisely, which is not done. So while it’s the type of experiment that’s been described, but as exactly as the samples are being processed and initial states, these are the quantities that, in my experience and my observation, are often missing*.”

P11

Another important aspect, which makes a detailed data description mandatory, is the reproducibility of results.

“*The circumstances and conditions that led to the data, if this is not somehow clearly shown or the experimenter himself did not think carefully and planned or even supervised, then you just have any data as a result and the next one does the same experiment with the same material and there comes out something completely different*.”

P11

In the field of materials science and engineering, reproducibility of experimental data is also an intrinsic problem, as it can arise from variation of measurement methods:

“*Even if you measure the same material (from same provider) in two different labs using the same device, the results might not be even close. Or sometimes the same type of device from different manufacturers will also give you data variation on the same material…*”

P8

### Results RQ2: Researchers as data generators

During their daily work, researchers take different roles, as they generate, reuse, or share research data. To take this into account, the interviewed researchers were asked, whether they generate research data, which is seen as a prerequisite to the analysis of data sharing practices. From the interviewees, six researchers stated to generate their own experimental data. In four cases, researchers contracted a third person for data generation. These cases are included, as it is assumed that this data could possibly be shared. One researcher explained:

“*We try to get the material characterised ourselves. Either we can do it locally or we have to visit someone, visit some labs to do experiments there or we have to send the material really to a company who does this job*”

P8

Moreover, 11 said to generate also computational data, e.g. the output files from conducted simulations.

The researchers were asked if their employer has a data management policy, prescribing them how to store their generated research data. This was the case for seven researchers, two of them stated to write data management plans for their generated data. On the contrary, four researchers stated that in absence of a data management policy they decide alone how to store their data.

### Results RQ3: Researchers as data reusers

**Fig 1:**
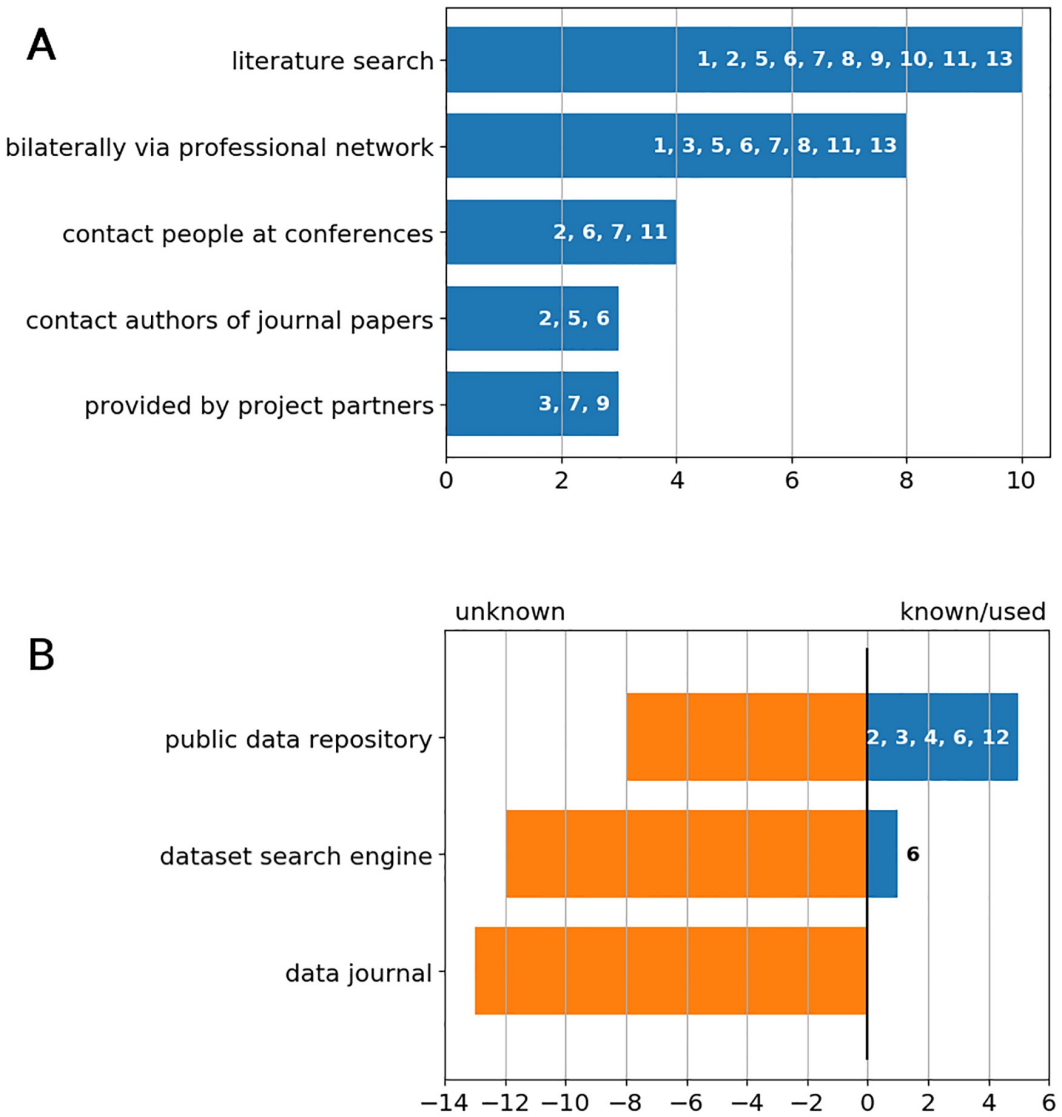
Methods for search for research data. A: used methods for search for/get access to research data (traditional). B: usage/knowledge on further methods for search for data (modern).

In the conducted interviews, a positive attitude towards data reuse is found, as all but one researcher stated to intend to reuse research data. The methods, which researchers use to search for data or gain access to data, are shown in Fig 1 in bar charts. Fig 1A summarises the use of methods, which the authors classify as traditional search methods (the numbers in the bars correspond to the number assigned to each participant). Most frequently used is the literature search. Two researchers mentioned to use software tools for data extraction from shown plots and two mentioned raw data provided as supplemental material. The remaining traditional search methods include gaining access bilaterally via the professional network, contacting people at conferences, contacting authors of journal papers or research data are provided by project partners.

In the interviews, the researchers were explicitly asked if they use any search method/resource, which the authors would name as modern search methods, see Fig 1B. These include public data repositories, dataset search engines and data journals. Public repositories were known or used by five researchers. In detail, three of them knew the “Zenodo.org” repository, one researcher actively searched at “Zenodo.org”, one downloaded data from an university repository and one used “Mendeley Data” to access data. Thus, less than the half of the interviewees were familiar with public data repositories at all. Out of 13 conducted interviews, only one researcher had heard of “Google Dataset Search”, but never used it. The concept of dataset search engines was unfamiliar to all other researchers. None of the interviewed researchers were aware of the existence of data journals.

In spite of the will of interviewees to reuse research data, there were several obstacles mentioned. As the needed data is very specific, findability is a big problem:

“*I think you need to take a lot of time to find the exact data that you need*.”

P10

“*It’s very rare actually to find the data you can use*.”

P6

Another aspect is the lack of standard repositories:

“*In this community, it’s not like there is a standard library or database, you just go there you can get all the information—it is like it is scattered everywhere. Everyone is measuring different things based on the interest and there is no common database for you to just search*.”

P8

Moreover, as the data generator often collects data for specific projects, the dataset might be incomplete for the usage for another purpose.

“*The problem is always that, there is often a lack of data that the dataset is complete and useful for my work*.”

P11

Due to the lack of standardised descriptions of research data, research data can be unusable because information were forgotten in the description:

“*Even if one finds one or the other in the literature, it is so that certain quantities are missing and if you ask then, of course, you will not find them after a few years, which is a pity*”

P11

Despite the fact that most researchers search in the literature to find research data, it was striking that 10 out of 13 interviewed researchers stated they had never seen a dataset citation. From the three researchers, who had seen data citations, two said that they were very rare.

### Results RQ4: Data sharing practices

**Fig 2:**
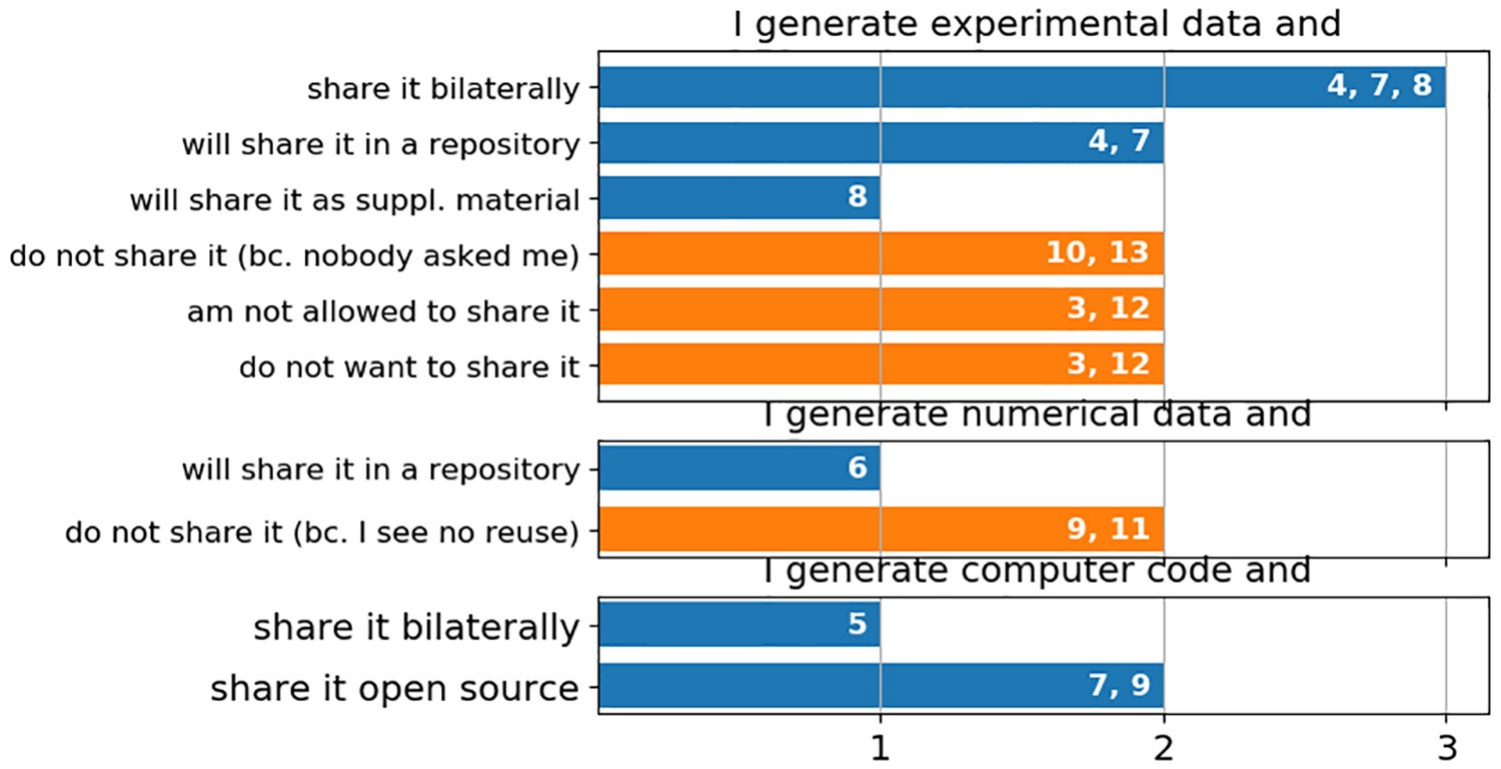
Data sharing practices of the interviewed researchers.

The interviewees were asked if they share the research data, which they generate. The answers are grouped with respect to the type of data, i.e. experimental data, computational data or computer code, and are summarised in Fig 2. Experimental data is currently shared only bilaterally. Two researchers stated they share in direct contacts with known persons, two stated they had shared data to contacts from conferences, one stated to have shared data via “Researchgate” and one shared with a contact per email. Thus, both the search for research data and the sharing is mostly organised via personal contacts. This trend might change, as three participants state that they plan to share experimental data in near future (as supplemental material to a journal article, or via an university repository). These participants stated to be affected by their funder’s data sharing policy.

Four researchers do not share their experimental data. The reason two of them gave was that until now nobody had asked them to do so. Probably these two see no fundamental problems in data sharing and might share their data, if asked by other researchers, publisher or funding bodies. In contrast, two researchers stated that they do not share their data, because they are not allowed and they do not want to share it. For researchers in this domain, legal restrictions for data sharing often arise from corporation with industry. One researcher also expressed additional concerns, e.g. fear of misuse/misinterpretation:

“*We always block that data because we do not want that anyone does anything with this data without any control. This is a must to avoid “nonsense production”. So I think that’s a bit dangerous*.”

P12

Regarding computational data, one researcher stated to share generated data soon in a public repository. This researcher also declared to be affected by the funder’s data sharing policy. Two researchers stated that their generated computational data (e.g. output files of computer simulations) is not of interest for others. Thus, they do not share it. This could actually be the case for more than two interviewees, as it was not separately asked for generated computational data, but for generated data in general.

Three researchers stated that computer code was an output of their scientific work. These three share their code, either bilaterally or as open source code. The positive attitude towards code sharing might be linked with the longer tradition of code sharing.

### Results RQ5: Incentives and obstacles to share research data

**Fig 3:**
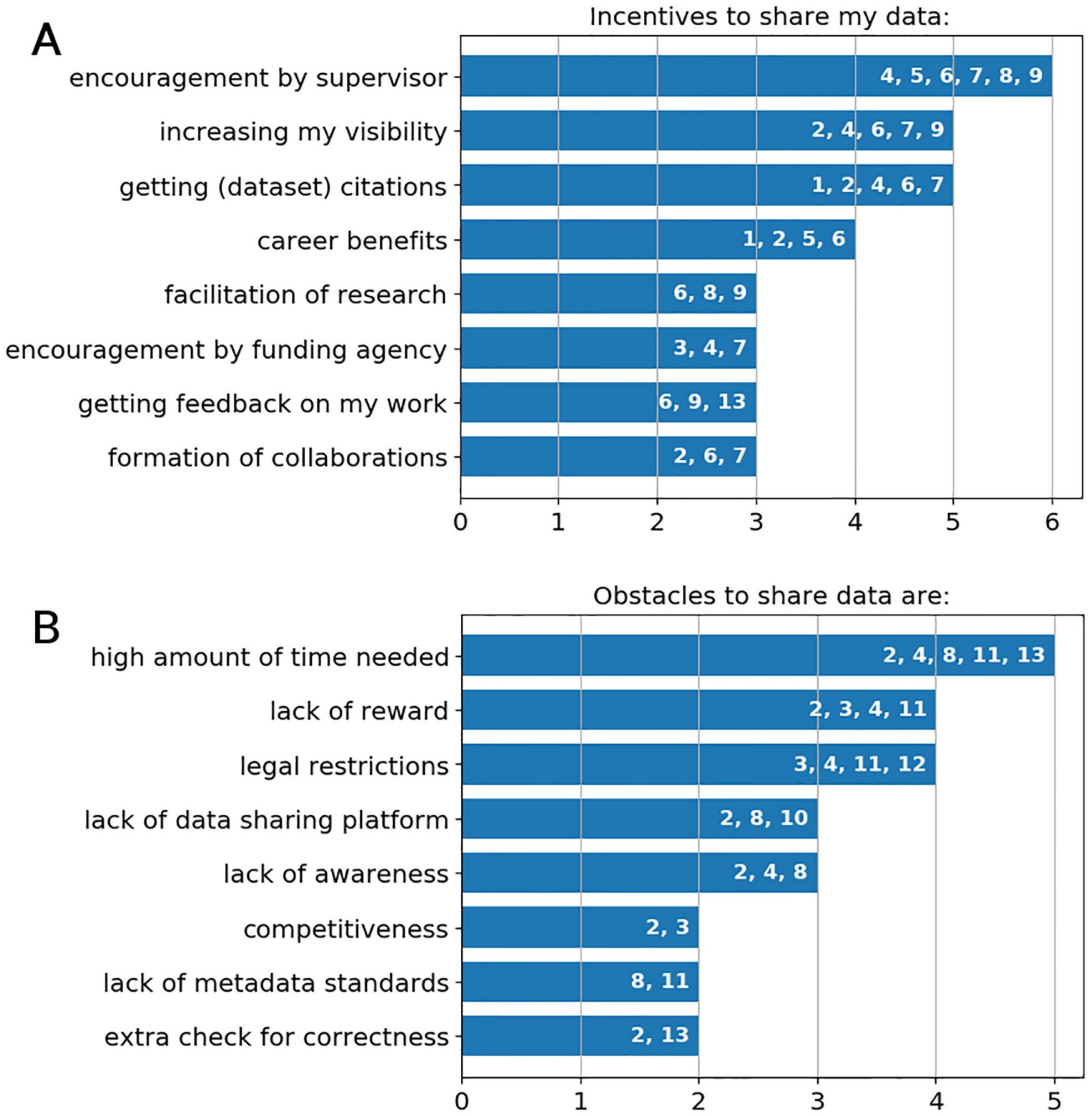
Incentives and obstacles to share research data. A: Incentives. B: Obstacles.

The participating researchers were asked what is (might be) an incentive to share their research data, see Fig 3A for a summary. Remarkably, all six researchers, who share their research data or computer code in any form, were encouraged to do so by their supervisor or employer. This encouragement was also the most frequently mentioned answer. Five interviewees, respectively, named an increase in their visibility as a researcher and being cited (either traditional or dataset citations) as possible motivation. General career benefits were possible incentives for four researchers. The following points were mentioned by three researchers each: facilitation of research in general, encouragement by funding agency, getting feedback on the own work and possible formation of new collaborations.

In the current study, the most frequently mentioned obstacle to share research data is the high amount of time needed to prepare, check and describe the data adequately, compare Fig 3B.

“*You need to spend time to prepare them in a way so that other people can understand what they are. Because raw data is usually messy, you need to label it, you need to make it tidy that other people can understand what is going on in your big data and sometimes we don’t have that time to spend and tidy up our data*.”

P4

Four researchers named a lack of rewards as an obstacle to share their data. Different aspects were mentioned:

“*I need to report my progress and these things to my line manager and to the university. I’m not sure if this (data sharing) is something I can report*.”

P4

“*(The) funding agency is again giving you funds when they look at your CV: how many papers you have published and so on. The criteria…They would promote the open data project, but at the same time they are looking how many papers you have published*.”

P2

“*It (data sharing) would also have to be rewarded in that respect (…) If they (data) are published in such a way that they are provided with a publication number, as on the level of a paper and that e.g. he can use it in his reference list and also in his list of publications, which is also credited him, in a dissertation—well, there is the PhD-thesis, but if he did it properly, this documentation of the data, then this is an additional effort and I would say, this should be rewarded*.”

P11

Legal restrictions were also named by four interviewees: as research projects were partly funded by industry, researchers saw an obvious conflict of interest to data sharing. Three researchers stated that a standard library or data sharing platform is missing for their scientific domain. This is a problem for data sharing, as well as for data search and reuse, as it was already discussed in the previous Sections. However, as the generated research data is very diverse, to find or create such a standard platform might not be easy.

A lack of awareness was named by three researchers as an obstacle to data sharing.

“*But I think the point is these things are never reaching to the most researchers. (…) And also, the point is, normally you should be reached supervisor level. If the supervisor doesn’t know, normally, the student will never know*.”

P8

Two researchers express serious concerns about Open Data regarding the competition between researchers.

“*The competition has such a high level now that people are more interested in doing science, but in a closed room, in a closed laboratory, they do not want to really, maybe, some of them, they do not really want to share too much their information, too much their data*.”

P2

“*On the one hand, we want to publish, what do I know, open to the public the models that we develop, also open to the public data that we generate, so that we parametrise the models. That’s all legitimate and sounds very well. On the other hand, we too, and everyone at the university, I believe, is in some competition with others. And there I have my fundamental problem. I’m not sure, if I want to do everything Open Access. Because I say we build know-how, over many years, skills. Do I want to share everything?*”

P3

While competition between researchers is inevitable, a clarifying discussion what exactly should be shared, e.g. measured data, developed models, methods or algorithms, could improve the acceptance of the Open Data idea among researchers.

The lack of metadata standards was already discussed with respect to data reuse, and obviously it hinders also data sharing, as it was stated by two researchers.

“*Again, that would need such a clear definition, a given structure, where somebody simply copies the data into the corresponding file and does not have to think about it oneself, because if he himself starts, somehow setting up a database structure for his data and everybody has a different one, then that’s too much of an effort to use that*.”

P11

As a last point, two researchers mentioned a particular need to do extra checks for correctness of their data before publishing (which then again is time consuming). With the data openly available, researchers might feel more exposed to criticism on their work.

### Results RQ6: Open Data concept—Familiarity, pro and contra

When asked if the terms Open Data and Open Science meant anything to them, three out of 13 researchers said they were not really familiar with them. Eight researchers said they had heard the terms before or had an idea what they meant, which could be attributed to the fact that most researchers are likely to make the connection to open access, even though they may have not come across the term before.

With regard to how researchers defined open data, it was interesting to see that they took different perspectives. While some thought of open data in terms of making their own data openly available, others viewed data sharing as something other researchers do that could potentially benefit them. 6 researchers said that open data was about “making data openly available”, “sharing data for free” etc. Some researchers added different aspects such as Open Data

“*generating added value for, maybe in other contexts too*.”

P1

“*These FAIR arguments that it has to be freely accessible, like it has metadata to understand the data*”

P7

“*Save a lot of money and a lot of time*.”

P10

“*The data should be accessible without every time you have to ask the author for the data*.”

P8

What was striking was that although the term had a positive connotation for most researchers, there was one researcher who stated that open data meant that “people I do not know can access my fundamental data”, i.e. the term had a negative connotation.

**Fig 4:**
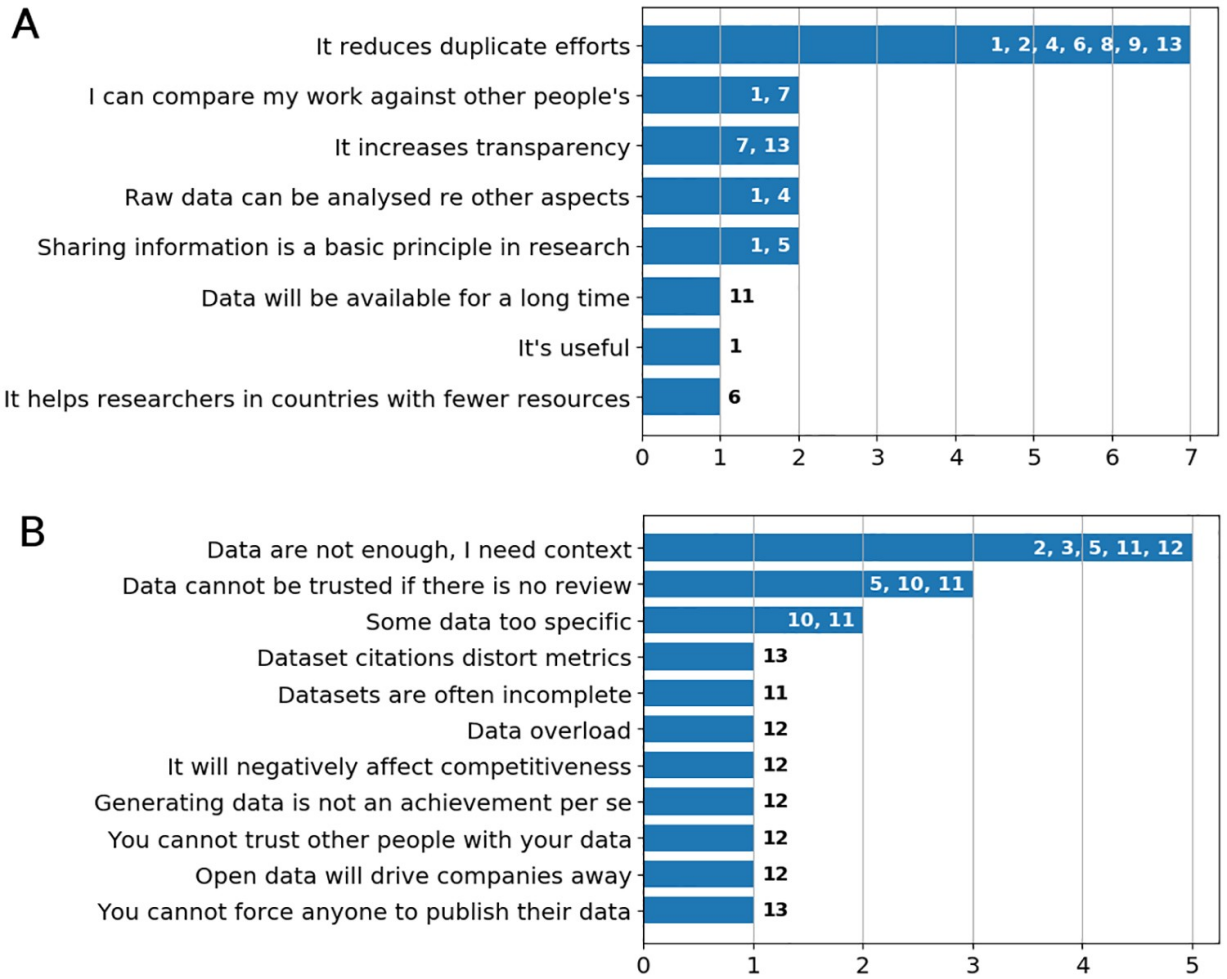
Open Data: Pro and contra. A: Pro. B: Contra.

Towards the end of the interview, researchers were asked for arguments in favour or against Open Data, see Fig 4. From the positive aspects, seven researchers mentioned that Open Data can reduce duplicate efforts for measurements/data generation. Other aspects, mentioned by two researchers each were the possibility to compare the own work with those from other researchers, an increase in transparency, the possibility to analyse published raw data with respect to other aspects and that sharing is a basic principle of science. Mentioned by one researcher each was that data will be available for a long time, that Open Data supports researchers in countries with fewer resources and a general usefulness.

Regarding negative aspects, the most frequently mentioned argument is the need for a detailed description of a dataset. As already addressed in the Section on data reuse, without sufficient context information shared research data is useless. The time needed for this detailed description was also the most frequently mentioned obstacle for data sharing. Three researchers stated that they do not trust datasets, when there is no review process. Some data might be too specific to justify the high amount of time needed for the preparation and description, as two researchers said. They suggested, this data might better be shared bilaterally. Several aspects were mentioned only once in the interviews: so could dataset citations distort citation metrics, lack of completeness of datasets might cause problems, generating dataset should not be seen as achievement per se and fear of data misuse was expressed. Moreover, concerns were raised that Open Data could produce a general “data overload”, have a negative effect on competitiveness or will drive companies away.

## Discussion

### Discussion RQ1: Diversity of domain specific research data does challenge open data

In the considered scientific domain, researchers stated they *need mostly experimental data* to conduct their research. This data is very diverse: used types of experiments and measured quantities strongly depend on the research focus. A detailed description of the experiments/devices/conditions under which data was generated is crucial for the reuse of this data and to ensure reproducibility of results. However, standardisations for such descriptions do not exist, not even for long used types of experiments. Most likely new research trends will generate/need different types of research data, possibly measured using newly developed devices. It can be expected that this will provide a big challenge for a research domain, which seems to be slow in developing standardised data description methods. The findings above fit well to the description of “small science” or the ‘‘the long tail’’ of science as described in [16].

### Discussion RQ2: Need for data management policies

11 out of 13 interviewees stated they *generate research data*, either experimental (including contracting third persons for measurements) or computational. Only those researchers, who generate their own research data, do have the possibility to share data. Seven interviewees were affected by data management policies of their employer, while four said that storage or their generated data is left to themselves. Even if these universities/research centres do not encourage data sharing, the danger of loosing data should motivate them to install data management policies.

### Discussion RQ3: Data findability might increase—Obstacles for reuse remain

*Data reuse* is intended by all but one interviewed researcher. The methods used to search for data or accessing data could be named as traditional: they include literature research as well as getting access bilaterally via the professional network, contacting people at conferences, contacting authors of journal papers or data being provided by project partners. Thus, in these search methods a direct contact between data reuser and data generator exist. These results are in accordance with findings of [10], who conducted a large scale domain specific analysis of search methods. Closest to the domain considered here might be the subject “Earth and Environmental science” where journals and personal exchanges are the two most frequently used methods.

Among search methods, which could be called modern, public data repositories are known to only five researchers (thus less than the half of the interviewees). [10] mentioned repositories and databases as the third most frequently used search methods of scientists in “Earth and Environmental science”. Thus, the investigated scientific domain might have some catch-up potential regarding repositories.

As already mentioned, dataset search engines a a very recent development. In the current study, one out of the 13 interviewees knew “Google Dataset Search” but never used it. Thinking of the important role of “Google Scholar” for searching traditional publications, “Google Dataset Search” might have a big potential to increase the findability of shared datasets across different storage possibilities.

Data journals are also a rather modern method both to search for or share data, but in the current study, none of the interviewees had ever heard of data journals. This was surprising for the authors, as for example Elsevier as a big publisher promotes co-submission between their data journal “Data in Brief” and approximately 1400 regular journals.

Although nearly all interviewed researchers had not heard of dataset search engines and data journals, several of them showed spontaneous interest and stated to check on these possibilities in the future. These interested researchers covered the whole range of the interviewees regarding age and career stage. As one researcher put it:

“*I am learning a lot in this interview*.”

P9

This could be an indication that methods used for research data search within this domain could change if information/teaching was provided. However, due to the small amount of conducted interviews the presented findings would need further validation. In general, the researchers’ positive attitude towards data reuse is in agreement with findings from [19]: “Expressed lack of trust in existing data and perceived norms against data reuse were not found to be major impediments for reuse contrary to our expectations.” Concerns as found in [20] “This study shows that scientists’ concerns about data reuse (e.g., misinterpretation and infringement) can negatively impact their reuse behaviours.”, were not expressed by the interviewees. Despite this positive attitude, several problems in data reuse remain. One big challenge is the findability of data, which might be overcome, e.g. by using dataset search engines. A second big challenge is the lack of standard repositories, which was mentioned related to data reuse but also as an obstacle for data sharing. Moreover, some more domain specific aspects can hinder data reuse. In the interviews, it was addressed that the data generator usually collects data/conducts measurements for a specific project. The data reuser might have a (slightly) different research focus, such that the data is incomplete for this purpose. Due to the lack of standardisation in the data description, data can be unsuitable for reuse, as some details are missing in the description. Also, this lack of a standardised data description also increases the time needed for data sharing, which is the most frequently mentioned obstacle for sharing data.

Despite the fact that most researchers search in the literature to find research data, it was striking that 10 out of 13 interviewed researchers stated they had never seen a dataset citation. From the three researchers, who had seen data citations, two said that they were very rare. These findings are in agreement with those of [23]. Training for scientists might help to improve this situation. When all reused datasets are cited correctly, this is likely to be an additional incentive to share research data.

### Discussion RQ4: Bilateral data sharing dominates currently—Influence of project funding rising

The *data sharing behaviour* of the interviewed researchers differed depending on the type of data. Experimental data is currently shared only bilaterally (in different forms). Thus, a personal contact between data generator and data reuser is ensured. However, three researchers stated to share their experimental data openly in the near future (as supplemental material to an article or in a university repository). These researchers were affected by their funder’s data sharing policy. This finding could be in contrast to [40], who found it very difficult to recover data that are required by the funder to be shared. However, this (possible) change in the sharing behaviour of researchers is too new to be assessed finally. From the researchers, who do not share their experimental data, two stated they never shared their data. These two seem to be undetermined, saying nobody ever asked for their data, such that it might be possible to convince them to share their data. On the contrary, two researchers stated that they are both not allowed to share their data and they do not want to share it. These aspects might need to be addressed in the literature in more detail. Many studies mention legal restrictions in scientific domains, which work with data from human subjects, e.g. [7, 17, 27]. In these scientific domains, training of the researchers on legal aspects (such as informed consent of the participants) could enable data sharing. In materials science and engineering, legal restrictions often arise from corporations with industry. Thus, in materials science and engineering it seems unlikely to overcome this restriction for data sharing. For the interviewed researchers, the project funding had a big influence: whether they plan to share their data openly (due to funding body policies) or whether sharing was in conflict with the interest of the industry involved in the funding.

Additional to the mentioned aspects, the two researchers mention explicitly that they do not want to share their data. In the literature, the big survey studies rely on the researchers willing to participate. Those, who have no interest in data sharing can be expected to be more likely to skip such surveys, [28]. This could be a key problem in obtaining results representative for all researchers. Moreover, in conducted surveys options like “I do not want to share my data” or “data sharing is not in my interest” are usually not provided, [17, 27]. This point could add interesting insights in future research.

Regarding the sharing of computational research data, one researcher stated to share data soon via a public repository, as he is affected by his funders data sharing policy. Two researchers stated that their generated computational data (output files of computer simulations) was not of interest for other researchers and therefore the data is not shared. This could be the same for more researchers, as 11 from them generate computational data, but only three of them commented on (not) sharing it. In the interviews, the question of data sharing was not asked separately for each type of data. The sharing of experimental data and computational data does not seem to obtain the same amount of attention from the researchers. A deeper discussion if or which shared computational data could be of interest for reuse could be needed.

All of the three researchers, which stated to generate computer code, do share their code: either bilaterally or as open source code. The positive attitude towards code sharing might be linked with the longer tradition of code sharing.

### Discussion RQ5: Encouragement is the best incentive and lack of time the biggest obstacle for data sharing

Regarding *incentive to data sharing* it is striking that all six researchers, who share their research data or computer code in any form, were encouraged to do so by their supervisor or employer. Some of the other named incentives were rather abstract and hard to quantify, e.g. increased visibility, career benefits or general facilitation of research. More concrete incentives were getting (dataset) citations, encouragement by funding agency, getting feedback on the own work and the possibility of new collaborations. In this study, encouragement of the supervisor, employer or funding agency is seen as the most successful tool for data sharing, as it is reported by those researchers who share already or plan to share their data soon. In the literature, incentives seem to receive slightly less attention than obstacles. For example, in Spinger’s whitepaper [27], challenges are explicitly investigated, incentives are not. To name another big and well cited study, [17], address possible incentives only in the literature review but not in their research questions. In the extensive literature review on data sharing, [7], named motivating factors are scientific progress, data sharing policies of funding agencies, reduction of costs, data sharing policies of publishers and safeguards against scientific fraud. Thus, the most frequently given incentive in the current study, encouragement by the supervisor or employer, is not present. In [41], publishers’ data sharing policies of 28 engineering journals were investigated. While most publishers supported data sharing, only few had strong policies, which make data sharing mandatory. As none of our interviewees mentioned journal data sharing policies as an incentive for data sharing, it seems that in the field of materials science and engineering publishers still have potential to further promote data sharing.

In this study, frequently mentioned *obstacles to data sharing* are high amount of time needed for a detailed data description, lack of rewards, legal restrictions, lack of a standard data sharing platform and lack of awareness. As mentioned before, the lack of a standard data sharing platform reduces the findability of data and a detailed data description is mandatory for data reuse, due to data diversity. The named obstacles are in accordance with findings of the literature review, [7]. Interestingly, [7] mentioned two other obstacles to be important. The need to have control over ones data, was not expressed explicitly in this study and the fear of data misuse was mentioned only by one interviewee of this study. Instead, interviewees also named competitiveness, lack of metadata standards and the need for an extra check of correctness as obstacles, which seems not to be reflected in the literature according to [7]. To promote Open Data, training on data sharing could help to remove the lack of awareness, as frequently mentioned in the literature, [7]. The lack of a specialised data sharing platform could be overcome, if the findability of datasets increase with the usage of dataset search engines. Data could be stored in general repositories and still be found, reused and cited. The mentioned lack of rewards is a more difficult topic: here universities could credit researchers/PhD students for shared data, but also funding agencies would need to honour the effort made by data sharing when deciding whose project gets funded. Obstacles which cannot be overcome are the amount of time needed for a proper description of research data, legal restrictions, competitiveness and lack of metadata standards. These obstacles will remain and can restrict the amount of shared data notedly.

### Discussion RQ6: Training on Open Data is crucial for acceptance

The Open Data concept was known to most of the interviewed researchers, only three of them stated to be unfamiliar with the term. The others could not precisely define the concept but mostly understood Open Data as making the own research data openly available for others. As discussed in the previous paragraph, training on data sharing would be very helpful for most scientists and might also clarify the meaning of the term Open Data for some attendees. The scientists saw as most positive aspect in Open Data that it has the potential to reduce duplicate efforts in data generation. Other positive aspects, such as an increase of transparency or using data for multiple purposes were mentioned by only one or two interviewees. When asked for negative aspects of Open Data, researchers stated that a simple sharing of raw data is not useful in their point of view. They stress that the data needs to be well documented, providing enough context, together with a review process for rating data as trustworthy are crucial for successful reuse of data. Two researchers believe that some data might be too specific to be shared. Other negative aspects are mentioned only once. The scientists’ need for precise data documentation and some verification (e.g. a review process) is well understandable. There are several ways how these points could be met: First, research data can be shared as supplemental material to a journal article, thus including the detailed description of experiments and providing a review process. Second, research data can be shared in a data journal (possibly accompanied by a classical research article in a partnering journal), in this way also a detailed description of the data and a review process is present. Third, when a classical research article is published (including the details on the experiments), the corresponding research data can be shared on a public repository. In this way, the additional effort for data description is reduced and the general scientific work is reviewed (although not the data itself). All these points could be addressed in a training on data sharing, which was already found a useful action before.

## Conclusions and outlook

In this study, detailed interviews were conducted on the current state of open research data practices of 13 researchers in the field of granular materials, which is part of materials science and engineering. The research data in this scientific field was found to be very diverse and often generated for a specific research focus. The interviewees stated that openly accessible research data would help them with their work, but they needed detailed descriptions on the data and the complete data generation process. According to [16], this is a typical case for “small science”, where “data tend to be small in volume, local in character, intended for use only by these teams, and are less likely to be structured in ways that allow data to be transferred easily between teams or individuals.” In current open research data practices, the interviewees mentioned problems regarding the findability of data. The lack of a standard library was seen as a problem, but this would be difficult to set up, given the diversity of the data. Researchers employed traditional search methods and were mostly unaware of dataset search engines, data journals or searching public data repositories. Thus, the findability of data might increase, when the mentioned methods are applied, but will meet its limits regarding the diversity of the generated and needed data. As mentioned above, a successful reuse is possible only when the data is described in high detail. The time needed for describing research data is again the main obstacle to data sharing. At this state, some of the interviewed researchers share their data bilaterally (but not openly) and all of them were encouraged to do so by their supervisor/employer or funding body. Some researchers stated to share their research data openly in near future, as they were affected by their funding body’s data policy. Those researchers who don’t share named the lack of the following as obstacles: time (for data description), rewards, awareness, and data publishing platform/standard library. Two researchers pointed out that not only legal restrictions prevent them from data sharing, but they do not want to share their data. These researchers mentioned competitive aspects and fear of data misuse as reasons. While open research data practices in general are subject of intense research, researchers who see data sharing contrary to their own interests might be underrepresented in many studies. Limitations of the current study are seen in the small number of interviewees as well as in the choice of interviewees from the authors’ professional network (as it was not possible to apply a purposeful sampling technique). Moreover, it was not possible to interview enough women to investigate gender related aspects. The obtained results are not transferable to different scientific domains.

From the described results and conclusions the following measures are suggested to promote Open Data.

### Actions to support Open Data

- training in open data practices can help researchers to
  1. find datasets more easily
  2. correctly reuse data via dataset citation, thus making data sharing attractive through getting citations
  3. share their own research data, such that
    - the effort for data description is minimised
    - others judge their data as trustworthy (e.g. because of review process)
    - long term accessibility of data is ensured and FAIR principles are met
- universities/research centers could
  1. install a data management plan to prevent research data from being lost
  2. think of rewarding scientists for data sharing, possibly similar as rewards for research papers
  3. encourage supervisors and students to get training in open data practices
- funding bodies could
  1. install a data sharing policy
  2. provide training/information on open data practices
  3. think of rewarding scientists for data sharing, possibly similar as rewards for research papers
- publishers could
  1. consider installing stronger data sharing policies
  2. further promote data sharing via data journals

### Remaining restrictions on Open Data (in this scientific domain)

The investigated scientific domain is materials science and engineering and is seen as an example of “small science”, [16]: the generated research data is very diverse, generated for a specific purpose and difficult to transfer between researchers. In “small science” Open Data most probably will stay less common than in “big science”, where “Data from big science (large teams, long-term projects, extensive instrumentation) may be great in volume but usually are consistent in structure.”, [16], and thus easier to transfer between researchers. In addition to this, in materials science and engineering legal restrictions for data sharing often arise when the funding of research projects involve industry. These legal restrictions can be expected to remain no matter which action researchers, universities or funding bodies take.

To give an example of the challenges in Open Data in materials science and engineering, imagine “researcher A” needs a specific dataset for his/her work. To avoid spending time and money for experiments, “researcher A” can consider reusing existing research data. The first question is, if another “researcher B” did generate exactly the needed data, as data is very diverse in this scientific domain. If so, “researcher B” must be allowed to share his/her data (i.e. no legal restrictions apply) and also be willing to share (little incentives exist). If so, “researcher B” must have the time to precisely describe how the data was generated (no metadata standard exists). If so, “researcher B” must choose a way for sharing the data, e.g. in a repository (no standard library exists and there is a lack of training for data sharing). If so, “researcher A” must find this dataset, due to the high diversity of data, the lack of a standard library and lack of training in dataset search, findability is a problem. If “researcher A” found the dataset, he/she must judge it as trustworthy, check the documentation for completeness such that the dataset can be reused for his/her own work. This scenario is not impossible, but it is clearly less likely than in other fields of research, e.g. in genomics or other “big sciences”.

Two further restrictions mentioned by the interviewees were independent from the scientific domain. First, the lack of time to share research data is an important obstacle for data sharing, as also described in the literature, [7]. Even if the effort for data description can be reduced if data is published alongside classical research papers, still time is needed for data preparation and description. Researchers need to give lectures, supervise students, engage in university administration, apply for research funding on top of the actual research, thus finding the time for data sharing might be difficult. The second restriction mentioned by two interviewees is that they do not want to share their research data. Although it is admittedly hard, the aspects that make scientists choosing not to share their research data might need to be investigated more thoroughly in future works.

## Supporting information

S1 Text Interview guideline. (PDF)

S2 Text Collection of used quotes in this paper. (TXT)
