# Supplementary material for: Search, reuse and sharing of research data in materials science and engineering—A qualitative interview study
Source: PLoS One. 2020 Sep 15;15(9):e0239216. doi: 10.1371/journal.pone.0239216 (PMC7491734; doi:10.1371/journal.pone.0239216)
Supplement: S1 Text — (PDF) [file pone.0239216.s001.pdf]

S1: Interview Guideline

Obtain written consent

This interview is done as part of a research project funded by the **Austrian Science fund (FWF).**
In general, FWF funds basic research, this project is part of a **special programme: Open Research Data Pilot**. The pilot aims to create role models and to gain experiences with open access to research data. It is envisioned that in line with the concept of Open Science open research data becomes the norm for all FWF projects in the future.
The title of our project is **"An Open Data Pilot for the validation of Discrete Element Models"**. It aims to **develop a methodology for the validation of a Discrete Element Model for railway ballast**. All necessary research data for the validation will be generated and shared openly.
One work package looks at the use of open data and data sharing practices among material researchers.

Motivation

I would like to ask you a few questions now. Don't worry if you have never heard of Open Data; we don't expect you to have any prior knowledge. If you have any questions or if something doesn't make sense to you, feel free to ask. You can interrupt me at any time.

Participants' background

- Gender? Age?
- What is your research discipline?
- What is your current role?
- Which country is your primary place of employment, i.e. where are you based?
- How many years of experience do you have as a researcher? (Career stage)
- Have you been involved in EU research projects before?
- Could you name the top 3 journals and conferences where you would like to see your research published?
- Are you familiar with the concept of open science/open research/open data?
- What does the term *open science* mean to you?
- What does the term *open research data* mean to you?

Data needs, data generation and storage

- What **type of data** do you need to do your research?
- What type of research data do you **generate**?
- Where do you store your data, i.e. data you have collected/generated?
- Does your employer or your boss have a policy on storing data, i.e. do they tell you where and how to secure your data?

Data collection, search and reuse

- **Where** do you get your data from?
  (Is the data available somewhere? How do you get access? Do you generate it yourself?)

- How do you identify organisations or people that may have data that could be useful to you?
  - Do you use **literature** to identify data owners/sources?

  - Do you use **search engines** (e.g. Google Dataset Search)?
    - Which keywords/search terms do you use when searching for these data?
  - Do you use your **personal network** to identify data owners/sources?
  - Do you know what a **data repository**, such as Zenodo, is? Do you use them?
  - Have you ever heard of **data journals** (e.g. *Elsevier Data in Brief, Springer Scientific Data*)
  - What about **dataset citations**? Have you come across one before? Have you ever cited a dataset? Are dataset citations an incentive for you to share your data?

Data sharing practices, incentives and obstacles

- Do you **share** your data with anyone?
  With whom? How?
- If not, why?
- Under which circumstances would you share your data? What would motivate you to do so?
- When you **publish a paper**, do you share the associated data?
  (As supplementary material, in a repository)
- Has anyone ever **contacted you** to ask for the data you used after they read one of your scientific papers?
- Does data sharing have any **benefits for you** or your career? Are there any other incentives?
- Do you have to share your data because your employer /the funding agency/your superior at work **requires** you to do so?

Open Data & Open Science

Funding agencies such as the European Commission with its H2020 programme and national funding agencies have been stepping up efforts to try to get researchers to share the data they generate in publicly funded projects. Several policies have been implemented at national and EU level to promote research data sharing. These initiatives are often referred to as open science or open data.

- Do you think that the policies I have mentioned before affect you as a researcher?
- Do you think open science is a good idea? Do you find it useful?
  - How can open data benefit your research projects?
